# Phenotypes, antibacterial-resistant profile, and virulence-associated genes of *Salmonella* serovars isolated from retail chicken meat in Egypt

**DOI:** 10.14202/vetworld.2020.440-445

**Published:** 2020-03-11

**Authors:** Amal Awad, Mayada Gwida, Eman Khalifa, Asmaa Sadat

**Affiliations:** 1 Department of Bacteriology, Mycology and Immunology, Faculty of Veterinary Medicine, Mansoura University, 35516, Egypt; 2 Department of Hygiene and Zoonoses, Faculty of Veterinary Medicine, Mansoura University, 35516, Egypt; 3 Department of Microbiology, Faculty of Veterinary Medicine, Matrouh University, Egypt

**Keywords:** antibacterial susceptibility, broilers meat, multidrug resistance, *Salmonella*, virulence

## Abstract

**Aim:**

The present study was designed to investigate the occurrence and distribution of *Salmonella* serotypes in chicken meat samples, and to explore the susceptibility of the strains to antimicrobials, as well as their virulence-associated genes.

**Materials and Methods:**

Two-hundred retail chicken meat samples from different shops, as well as 25 stool specimens from retail shop workers, were included in the study. The collected samples were examined bacteriologically for the presence of salmonellae. *Salmonella* isolates were serotyped using a slide agglutination test for O and H antigens and were screened for the presence of five virulence genes (*stn, pef, inv* A *, sop* B , and *avr*A) using a uniplex polymerase chain reaction assay and for their susceptibility to 18 antimicrobial agents using the disk diffusion method.

**Results:**

Thirty-one *Salmonella* isolates belonging to 12 different serovars were identified. *Salmonella* Enteritidis and *Salmonella* Kentucky were the dominant serovars (22.6% each). *Salmonella* isolates displayed a high antibiotic resistance against erythromycin, sulfamethoxazole/trimethoprim, doxycycline, cephalexin, cefaclor, tetracycline, polymyxin B, cefuroxime, vancomycin, and streptomycin. All *Salmonella* isolates exhibited multidrug resistance (MDR) and demonstrated different virulence genes. The majority of *Salmonella* serovars (87.1%) harbored *sopB* gene, 54.8% carried *avr*A and *pef* genes, while all isolates carried *inv*A and *stn* genes.

**Conclusion:**

The presence of virulent MDR Salmonellae in raw chicken meat could allow the possibility of transmission of these resistant serovars to humans. Therefore, strict hygienic measures should be followed on the whole poultry production chain to decrease the potential transmission of *Salmonella* infection from poultry meat to humans.

## Introduction

Foodborne pathogens such as *Salmonella* (S) *enterica* species represent an important worldwide public health problem. *Salmonella* remains one of the major zoonotic bacterial foodborne pathogens, resulting in 93.8 million annual cases among patients having gastroenteritis, with an estimated 155,000 deaths each year [1]. Poultry is considered the main reservoir for a significant number of infections with *S. enterica* species, involving cross-contamination events at both the farm and retail levels [2]. A diverse incidence of salmonellosis has been reported recently [3]. The clinical manifestations of the disease among human patients ranged from self-limiting gastroenteritis to severe invasive meningitis, septicemia, and osteomyelitis [4]. The majority of cases are caused by the fecal contamination of foods of animal origin or the consumption of poultry or its products [5].

Monitoring *Salmonella* infection on poultry farms is a challenging problem and relies mainly on the application of biosecurity measures in the farm setting and the use of antibacterial agents [6]. However, the excessive use of antibacterial medications in the poultry industry, either as growth promoters or to treat disease, could result in the emergence of antibacterial resistance among *Salmonella* isolates with potential health hazards [7]. Multidrug-resistant (MDR) bacteria from poultry origins have the potential to transfer to humans through the food chain, a danger that has triggered serious public concern [8].

The degree of the pathogenicity of *Salmonella* infections in the host cell has been attributed to the presence of virulence genes which have a significant role in the occurrence of systemic infections [9]. The presence of the *inv*A gene in *Salmonella* pathogenicity islands is associated with epithelial invasion and the production of proteins of the type III secretion system (T3SS) [10], while the *stn* gene mediates enterotoxin production and is often associated with acute gastroenteritis [11]. The plasmid-encoded fimbriae (*pef*) mediates intestinal adhesion in certain *Salmonella* serotypes [12], while the multiple function effector proteins encoded by the *avr*A and *sop*B genes in the T3SS could facilitate endothelial uptake and invasion [13]. Therefore, recognizing the existence and characterization of *Salmonella* in retail chicken meat is important for emerging effective treatment approaches to control salmonellosis.

Currently, it is difficult to assess the impact of retail chicken meat MDR on public health due to the limited availability of data in the study area. MDR in retail chicken meat is not systematically monitored in most Egyptian governorates, and the existing data are scarce.

The present study was designed to investigate the occurrence and distribution of *Salmonella* serotypes in chicken meat samples, and to explore the susceptibility of the strains to antimicrobials, as well as their virulence-associated genes

## Materials and Methods

### Ethical approval

This study did not need ethical approval. However, the procedures performed in the current study were in accordance with the ethical standards of the institutional and/or national research committee and those of Mansoura University.

### Samples collection

A total of 200 samples were collected from 100 chicken carcasses (two samples from each carcass) from local retail chicken meat shops at Mansoura city, Dakahlia Province, Egypt, during the period between September 2017 and December 2017. In addition, 25 stool specimens were collected from retail shops workers in sterile cups. For chicken meat, the samples were collected separately in sterile polyethylene bags to prevent cross-contamination and kept in clean sterile containers to be transferred within an hour through insulated coolers containing cold packs bag to the laboratory for *Salmonella* isolation and identification.

### Bacteriological examination

The procedures of *Salmonella* isolation and identification were performed, according to standard methods (ISO6579:2002; International Organization for Standardization 2002). Briefly, 25 g from each chicken meat sample were cut into small pieces by sterile scissors. Then add to a stomach bag containing 225 mL sterile Buffered Peptone Water (BPW) (Oxoid, Hampshire, England). For the stool samples, 1 g was added to 9 ml BPW, then all the samples were incubated at 37°C for 18 h. 0.1 mL of the pre-enriched broth was transferred to 10 mL of Rappaport-Vassiliadis broth (Oxoid, Hampshire, England) and incubated at 42°C for 24 h. A loopful of the enriched broth was then streaked on xylose lysine desoxycholate agar and incubated for 24 h at 37°C. Biochemical examination of the suspected *Salmonella* colonies was performed, according to the (ISO6579:2002; International Organization for Standardization 2002) guidelines [14].

### Serotyping

Serotyping of the biochemically identified *Salmonella* isolates was accomplished according to the Kauffmann–White–Le Minor technique on the basis of surface antigen identification using polyclonal antisera (Difco Laboratories, Detroit, USA) to determine the O (somatic) and H (flagellar) antigenic epitopes at the Department of Food Hygiene and Control, Faculty of Veterinary Medicine, Benha University.

### Molecular identification of *Salmonella* virulence-associated genes

Genomic DNA was extracted from the identified *Salmonella* serovars, according to Ramadan *et al*. [15]. The polymerase chain reaction (PCR) assays were conducted in individual reactions using an applied Biosystems 96-well Thermal Cycler to detect five *Salmonella* associated virulence genes *(stn, pef, inv* A *, sop*B, and *avr*A). The sets of primer sequences and corresponding amplicon sizes are illustrated in Table-1. All PCR reactions were performed in a total volume of 25 μL consisting of 12.5 μL of 2×PCR master mixes (Promega, Madison, USA), 6 µL DNA templates, and 1 μL of each primer (Metabion, Germany) brought to 25 μL using DNA/RNA free water. PCR reactions and thermal conditions used were performed, according to the referenced authors (Table-1) [9,16,17]. An aliquot of each amplified product was subjected to electrophoresis in a 1.5% agarose gel. The separated bands were visualized and photographed under an ultraviolet transilluminator.

**Table-1:** Primers sequences used in PCR for detection of virulence genes in *Salmonella* serovars.

Primer	Sequence	Virulence factor	Amplified product	References
*stn*	TTG TGT CGC TAT CAC TGG CAA CC	Enterotoxin/Chromosome	617 bp	[9]
	ATT CGT AAC CCG CTC TCG TCC			
*Pef*	TGT TTC CGG GCT TGT GCT	Plasmid encoded fimbriae/Plasmid	700 bp	
	CAG GGC ATT TGC TGA TTC C			
*inv*A	GTGAAATTATCGCCACGTTCGGGCAA	*Salmonella* species/SPI-1	284 bp	[16]
	TCATCGCACCGTCAAAGGAACC			
*sop*B	TCA GAA GRC GTC TAA CCA CTC	Translocated effector protein/SPI-5	517 bp	[17]
	TAC CGT CCT CAT GCA CAC TC			
*avr*A	CCT GTA TTG AGC GTC TGG	Effector protein/SPI-1	422 bp	
	AGA GCT TCG TTG AAT GTC C			

PCR=Polymerase chain reaction

### Antibacterial susceptibility testing

*Salmonella* serovars were tested for their antibiotic susceptibility against 18 different antimicrobial agents belonging to different antimicrobial classes through the disk diffusion method following Clinical and Laboratory Standards Institute guidelines [18]. The following antibiotic disks (Oxoid, Basingstoke, Hampshire, UK) were used: Polymyxin B (PB; 300 μg), azithromycin (AZM; 15 μg), cephalexin (CL; 30 μg), cefuroxime (CXM; 30 μg), cefaclor (CEC; 30 μg), erythromycin (E; 15 μg), sulfamethoxazole/trimethoprim (SXT; 23.75/1.25 μg), streptomycin (S; 10 μg), neomycin (N; 30 μg), vancomycin (VA; 30 μg), norfloxacin (NOR; 10 μg), doxycycline (DO; 30 μg), *penicillin* (P; 10 μg), gentamycin (CN; 10 μg), tetracycline (TE; 30 μg), amoxicillin (AX; 25 μg), AX/clavulanic acid (AMC; 30 μg), and rifampin (RA; 5 μg). The isolates were defined as MDR if they exhibited resistance to three or more antimicrobial classes.

## Results

Out of the total examined chicken samples (n=200), 31 (15.5%) *Salmonella* isolates were recovered. None of the examined workers stool samples’ yielded positive results. The identified serogroups among *Salmonella* isolates were B, C1, C2, C3, and D. Group C3 was the predominant group in the obtained isolates (Table-2). Twelve serotypes were identified including, *Salmonella* Enteritidis (n=7), *Salmonella* Kentucky (n=7), *Salmonella* Typhimurium (n=6), *Salmonella* Molade (n=2), *Salmonella* Labadi (n=2), *Salmonella* Takoradi (n=1), *Salmonella* Papuana (n=1), *Salmonella* Tamale (n=1)*, Salmonella* Larochelle (n=1), *Salmonella* Infantis (n=1), *Salmonella* Inganda (n=1), and *Salmonella* Bargny (n=1). The *Salmonella* isolates demonstrated a high resistance against E (96.78%), followed by SXT (93.55%), DO and CL (93.55%), CXM (83.33%), CEC (87.19%), S (80.65%), PB (83.33%), TE (83.88%), P (70.97%), AX (67.8%), AMC (83.88%), VA (83.88%), RA (70.97%), and AZM (58.07%) (Table-3). In contrast, all recovered *Salmonella* isolates displayed a high sensitivity to NOR (100%), CN (96.77%), and N (83.87%). MDR (resistance to three or more antimicrobials) was observed in all tested isolates (Table-4).

**Table-2:** Distribution of *Salmonella* serovars in retail chicken meat samples.

Identified serotypes	Number of serotypes (31)	Prevalence %	Group	Antigenic structure	

				O	H
*Salmonella* Kentucky	7	22.6	C3	8,20	i:Z6
*Salmonella* Enteritidis	7	22.6	D1	1,9,12	g,m:-
*Salmonella* Typhimurium	6	19.4	B	1,4,5,12	i:1,2
*Salmonella* Labadi	2	6.5	C3	8,20	d:Z6
*Salmonella* Molade	2	6.5	C2	8,20	Z10:Z6
*Salmonella* Larochelle	1	3.2	C1	6,7	e,h:1,2
*Salmonella* Takoradi	1	3.2	C2	8,20	i:1,5
*Salmonella* Tamale	1	3.2	C3	8,20	Z29:e,n,Z15
*Salmonella* Papuana	1	3.2	C1	6,7	r,e,n,z15
*Salmonella* Infantis	1	3.2	C1	6,7	r:1,5
*Salmonella* Inganda	1	3.2	C1	6,7	Z10:1,5
*Salmonella* Bargny	1	3.2	C3	8,20	i:1,5

**Table-3:** Percentages of antimicrobial resistance among *Salmonella* serovars.

Antibiotic used (µg/disc)	Disk code	Sensitivity (%)	Resistant (%)
Erythromycin (15 μg)	E	3.22	96.78
Azithromycin (15 μg)	AZM	41.93	58.07
Cephalexin (30 μg)	CL	6.45	93.55
Cefuroxime (30 μg)	CXM	16.12	83.33
Cefaclor (30 μg)	CEC	12.90	87.19
sulfamethoxazole trimethoprim, (23.75/1.25 μg)	SXT	6.45	93.55
Doxycycline (30 μg)	DO	6.45	93.55
Tetracycline (30 μg)	TE	16.12	83.88
Polymyxin B (300 μg)	PB	16.12	83.33
Streptomycin (10 μg)	S	19.35	80.65
Gentamycin (10 μg)	CN	96.77	3.23
Neomycin (30 μg)	N	83.87	16.13
Pencillin G (10 μg)	P	29.03	70.97
Amoxacillin (25 μg)	AX	32.26	67.8
Amoxacillin/clavulanic acid (30 μg)	AMC	16.12	83.88
Norfloxacin (10 μg)	NOR	100	0.00
Rifampin (5 μg)	RA	29.03	70.97
Vancomycin (30 μg)	VA	16.12	83.88

E=Erythromycin, AZM=Azithromycin, CL=Cephalexin, CXM=Cefuroxime, CEC=Cefaclor, SXT=Sulfamethoxazole/trimethoprim, DO=Doxycycline, TE=Tetracycline, PB=Polymyxin B, S=Streptomycin, CN=Gentamycin, N=Neomycin, P=Penicillin, AX=Amoxicillin, AMC=AX/clavulanic acid, NOR=Norfloxacin, RA=Rifampin, VA=Vancomycin

**Table-4:** Virulence genes and antibiotic resistance profiles of *Salmonella* serovars.

Pattern	Serovars	Virulence genes	Antimicrobial resistance profile
1	*S.* Kentucky	*stn*, *pef*, *inv*A	TE, CXM, VA, E, PB, CEC, S, STX, P, CL
2	*S*. Kentucky	*sop*B, *stn*, *inv*A	RA, CXM, VA, E, PB, CEC, DO, S, AMC, STX, AX, P, CL
3	*S*. Kentucky	*sop*B, *stn*, *inv*A	CN, TE, CXM, E, AZM, CEC, DO, S, AMC, ATX, P, CL
4	*S.* Kentucky	*sop*B, *stn*, *inv*A	RA, CXM, VA, E, AZM, PB, CEC, DO, S, AMC, STX, AX, P, CL
5	*S*. Kentucky	*sop*B, *stn*, *avr*, *pef*, *inv*A	CN, RA, TE, CXM, VA, E, AZM, PB, CEC, DO, S, AMC, STX, AX, P, CL
6	*S*. Kentucky	*sop*B, *stn*, *avr*, *pef*, *inv*A	N, TE, CXM, PB, DO, STX, CL
7	*S*. Kentucky	*sop*B, *stn*, *pef*, *avr*, *inv*A	CN, N, RA, TE, CXM, VA, E, AZM, PB, CEC, DO, AMC, STX, AX, P, CL
8	*S*. Enteritidis	*sop*B, *stn*, *avr*, *pef*, *inv*A	RA, TE, CXM, VA, E, AZM, PB, CEC, DO, S, AMC, STX, AX, P, CL
9	*S*. Enteritidis	*sop*B, *stn*, *avr*, *inv*A	RA, TE, VA, E, PB, CEC, DO, S, AMC, AX, P, CL
10	*S*. Enteritidis	*sop*B, *stn*, *pef*, *inv*A	CN, RA, TE, CXM, VA, E, PB, CEC, DO, S, AMC, STX, AX, CL
11	*S*. Enteritidis	*sop*B, *stn*, *pef*, *inv*A	TE, CXM, VA, E, AZM, PB, CEC, DO, S, AMC, STX, AX, CL
12	*S*. Enteritidis	*sop*B, *stn*, *avr*, *inv*A	TE, CXM, E, AZM, PB, CEC, DO, S, AMC, STX, AX, CL
13	*S*. Enteritidis	*sop*B, *stn*, *avr*, *pef*, *inv*A	N, RA, TE, CXM, VA, E, PB, CEC, DO, S, AMC, STX, AX, P, CL
14	*S*. Enteritidis	*sop*B, *stn*, *avr*, *pef*, *inv*A	RA, TE, CXM, VA, E, AZM, PB, CEC, DO, S, AMC, STX, AX, P, CL
15	*S*. Typhimurium	*sop*B, *stn*, *pef*, *inv*A	TE, VA, E, DO, AMC, STX, P, CL
16	*S*. Typhimurium	*sop*B, *stn*, *pef*, *avr*, *inv*A	TE, CXM, VA, E, S, STX, P
17	*S*. Typhimurium	*sop*B, *stn*, *inv*A	RA, TE, CXM, E, AZM, PB, CEC, DO, S, AMC, STX, CL
18	*S*. Typhimurium	*sop*B, *stn*,* avr*, *inv*A	RA, TE, VA, E, PB, CEC, DO, S, AMC, AX, P, CL
19	*S.* Typhimurium	*sop*B, *stn*, *avr*, *invA*	RA, CXM, VA, E, AZM, PB, CEC, DO, S, AMC, STX, AX, P, CL
20	*S.* Typhimurium	*stn*, *pef*, *inv*A	CN, RA, TE, CXM, VA, E, AZM, PB, CEC, DO, S, AMC, STX, AX, P, CL
21	*S*. Labadi	*sop*B, *stn*, *inv*A	RA, TE, CXM, VA, E, AZM, PB, CEC, DO, S, AMC, STX, AX, P, CL
22	*S*. Labadi	*sop*B, *stn*, *avr*, *pef*, *inv*A	CN, N, RA, TE, CXM, VA, E, PB, CEC, DO, S, STX, AX, CL
23	*S.* Molade	*sop*B, *stn*, *inv*A	RA, TE, CXM, VA, E, AZM, CEC, DO, S, AMC, STX, CL
24	*S*. Molade	*sop*B,* stn*, *avr*, *pef*, *inv*A	RA, TE, CXM, VA, E, AZM, PB, CEC, DO, S, AMC, STX, AX, P, CL
25	*S*. Inganda	*sop*B,* stn*, *avr*, *inv*A	RA, TE, CXM, VA, E, AZM, PB, CEC, DO, S, STX, CL
26	*S*. Bargny	*sop*B,* stn*, *avr*, *pef*, *inv*A	RA, TE, CXM, VA, E, PB, CEC, DO, AMC, STX, AX, CL
27	*S.* Larochelle	*stn*, *inv*A	RA, TE, CXM, VA, E, PB, DO, S, AMC, STX, P, CL
28	*S.* Takoradi	*sop*B,* stn*, *pef*, *inv*A	VA, E, PB, CEC, DO, S, AMC, STX, P, CL
29	*S.* Tamale	*sop*B, *stn*, *avr*, *inv*A	E, PB, CEC, DO, AMC, AX, P
30	*S*. Papuana	*sop*B, *stn*, *avr*, *pef*, *inv*A	RA, TE, CXM, VA, E, AZM, CEC, DO, S, AMC, STX, AX, P, CL
31	*S*. Infantis	*stn*, *inv*A	CN, N, RA, TE, CXM, VA, E, AZM, PB, CEC, DO, AMC, STX, AX, P, CL

*S.* Kentucky=*Salmonella* Kentucky, *S.* Enteritidis=*Salmonella* Enteritidis, *S.* Typhimurium=*Salmonella* Typhimurium, *S.* Labadi=*Salmonella* Labadi, *S.* Molade=* Salmonella* Molade, *S.* Inganda=*Salmonella* Inganda, *S.* Bargny=*Salmonella* Bargny, *S.* Larochelle=*Salmonella* Larochelle, *S.* Takoradi=*Salmonella* Takoradi, *S.* Tamale=*Salmonella* Tamale, *S.* Papuana=*Salmonella* Papuana, *S.* Infantis=*Salmonella* Infantis, TE=Tetracycline, CXM=Cefuroxime, VA=Vancomycin, E=Erythromycin, PB=Polymyxin B, CEC=Cefaclor, S=Streptomycin, STX=Trimethoprim/sulfamethoxazole, P=Penicillin, CL=Cephalexin, RA=Rifampin, DO=Doxycycline, AMC=AX/clavulanic acid, CN=Gentamycin, AZM=Azithromycin, N=Neomycin

All *Salmonella* isolates were screened by uniplex PCR for the identification of virulence-associated genes (*inv*A, *stn*, *sop*B, *avr*A, and *pef*) and all were positive for at least two of the screened genes. Both *inv*A and *stn* genes were detected in 100% of the tested isolates, while *sopB* was harbored by 27 isolates (87.1%) and both *avr*A and *pef* were found in 17 isolates (54.8%). Interestingly, *S*. Enteritidis and *S*. Kentucky harbored high percentages of the virulence genes (Table-4).

## Discussion

*S. enterica* infections are considered an economically relevant disease in the poultry industry in Egypt and a serious public health potential worldwide. Currently, there are over 2500 recognized *Salmonella* serovars [19]. In the present study, 15.5% (31/200) of the examined chicken meat samples were *Salmonella* positive. Similar results were previously reported from Central Ethiopia [20].

The frequency of *S*. Typhimurium in poultry meat has been reported previously in Egypt from Assiut city markets [21] and from Mansoura city [22]. In the former study, the authors have isolated *Salmonella* from 44% of the examined raw chicken meat; while in the latter study, the authors have isolated different *Salmonella* serotypes (including *S*. Typhimurium, *S*. Kentucky, *S*. Molade, and *S*. Bargny) from processed chicken in a percent of 25.6% (30/120). On the other side, Tarabees *et al*. [23] isolated *S*. Enteritidis from 2% (2/100) and *S*. Typhimurium from 3% (3/100) of chicken meats. In contrast, a higher prevalence of *Salmonella* contamination was recorded in South Thailand (67.5%) [24] and China (54.0%) [25]. According to a meta-analysis of European Published surveys, the incidence of *Salmonella* spp. in poultry meat commercialized in Europe is estimated to be 7.10% (95% confidence interval: 4.60-10.8%) [26]. The diversity in the prevalence rates of *Salmonella* from retail chicken meat could reflect the differences in the level of hygiene followed during chicken handling and processing, the sampling time of the year, the sampling design, as well as diagnostic methods followed. The predominant *Salmonella* serovars identified in the present study were *S*. Enteritidis (n=7), *S*. Kentucky (n=7), and *S*. Typhimurium (n=6). Similar findings were previously reported in Egypt [22,27]. In the latter study, 77.4% (24/31) of the *Salmonella* isolates belonged to serogroups C and B, aligning with results previously reported by Abdel-Maksoud *et al*. [28]. In the current study, seven isolates were serotyped as *S*. Enteritidis. According to the European Food Safety Authority report, approximately 74% of human zoonotic salmonellosis is caused by *S*. Enteritidis and *S*. Typhimurium [29]. It is imperative to discover the most common serotypes in the examined raw chicken meat to determine the point (s) of contamination at different stages during slaughtering, scalding, defeathering, evisceration, processing and handling, particularly in low hygienic poultry retail outlets.

The antibacterial susceptibility testing revealed that the identified *Salmonella* serovars were sensitive to NOR (100%), CN (96.77%), and N (83.87%). Nearly, similar data were previously reported [30], while 83.88% of the obtained *Salmonella* serovars were resistant to AMC and 96.78% were resistant to E. Several studies have demonstrated that various patterns of antibacterial resistance exist in *Salmonella* isolates [27,30]. A high rate of MDR was found against most of the commonly used antibiotics. These findings should alert the farm owners to use alternative medications to combat the bacteria instead of using the traditional antibiotics against which bacterial resistance has become common. The current findings are similar to those previously reported in Spain and Turkey [31,32], and MDR *Salmonella* isolates from poultry origin have been reported worldwide [4,33,34]. The pattern of MDR observed in the present study highlights the drawbacks of the uncontrolled use of antimicrobials, which contributes to increasing the incidence of resistant pathogens among human infections. Hence, a continuous surveillance of antimicrobial resistance of zoonotic bacterial strains such as *Salmonella* spp. isolated from food and production environments will contribute to a successful control program.

The virulence of the obtained *Salmonella* strains is associated with a series of genes which is responsible for invasion, colonization, and spread within the infected host. The present study demonstrated various virulence genes among *Salmonella* serovars. For example, the *inv*A and *stn* genes were harbored by 100% of *Salmonella* serovars, which are in agreement with Osman *et al*. [35] and Ahmed *et al*. [36]. It has been suggested that the *inv*A gene is a specific marker for the molecular detection of *Salmonella* serotypes, while the *stn* gene is responsible for enterotoxin production [36]. In the current study, the prevalence of the *sop*B gene was 87.1%. This gene plays a significant role in the occurrence of diarrhea through activating secretory pathways or facilitating inflammation and altering ion balances within cells [36]. The *avr*A gene was detected in 54.8% of the obtained isolates. Other reports have indicated that this gene was only observed in serovars that cause severe salmonellosis in humans [37]. The *pef* gene was also identified in 54.8% of the isolates, highlighting the significance of fimbriae in the infection process. A similar detection rate (68%) was previously reported by Hudson *et al*. [37]. In contrast, a low frequency (6.7%) of the *pef* gene in *Salmonella* isolates was previously detected [36]. Interestingly, none of the examined stool samples yielded positive results, notwithstanding the fact that the risk of contracting the infection from contaminated products cannot be excluded.

## Conclusion

Our findings provide evidence for the presence of MDR *Salmonella* serovars with a wide distribution of virulence genes in chicken meat. These findings raise the suspicion that retail chicken meat often harbors zoonotic *Salmonella* serovars, representing a potential public health hazard. The possibility of transmission of these MDR serovars to humans requires regular monitoring of the production chain and amended farming practices, with particularly strict regulation of antibiotic usage.

## Authors’ Contributions

AA designed the study, conducted the experiments, and revised the manuscript, MG wrote the manuscript and participated in carrying out experiments, and EK and AS shared in performing the experiments and reviewing the manuscript. All authors read and approved the final manuscript for publication.
